# Interobserver Reliability of Schatzker, AO Foundation-Orthopaedic Trauma Association, and Luo Classifications for Tibial Plateau Fractures: Does Three-Dimensional CT Improve Outcomes?

**DOI:** 10.7759/cureus.22227

**Published:** 2022-02-15

**Authors:** Panagiotis T Masouros, George Mitrogiannis, Georgia Antoniou, Christina Chatzidaki, Dimitrios Kourtzis, Christos Garnavos

**Affiliations:** 1 Orthopaedics, Evangelismos General Hospital, Athens, GRC

**Keywords:** interobserver reliability, ao-ota classification, luo, schatzker, classification, tibial plateau fracture

## Abstract

Objective

To assess the interobserver agreement of the most widely used classification systems (Schatzker, AO Foundation-Orthopaedic Trauma Association (AO-OTA), and Luo) and investigate the impact of multiplane CT scans on their reliability.

Methods

Twelve raters (seven consultants and five senior trainees) were invited to classify 25 cases of tibial plateau fracture randomly selected out of a large database. Initially, they were asked to classify the fracture according to Schatzker, AO-OTA, and Luo based on plain anteroposterior (AP) X-ray and axial CT images. This procedure was applied for 25 cases consecutively. Next, the raters are given access to the multiplanar CT views of the same cases and were requested to reclassify each case. The interobserver agreement was calculated using the Fleiss kappa coefficient.

Results

An overall fair inter-rater agreement was observed for the Schatzker classification based on the plain AP X-ray (k=0.361) with a slight improvement after three-dimensional (3D) plane CT views (X-ray: k=0.361; 3D CT: k=0.364). For the AO-OTA classification, the relevant values were 0.204 and 0.231 based on plain X-ray and multiplanar CT, respectively. Finally, the Luo classification achieved the highest scores among the three classification systems (k=0.498), but its inter-rater agreement can still be characterized as moderate. No statistically significant improvement in the interobserver agreement was found for any classification even if only the consultants’ subgroup was included in the data analysis.

Conclusion

All three classification systems failed to achieve a substantial agreement among the raters, with only a nonsignificant improvement after providing advanced imaging. This finding reflects the intrinsic weaknesses of the classification systems themselves rather than the disagreement on the fracture pattern due to unsatisfactory imaging.

## Introduction

Tibial plateau fractures are characterized by significant heterogeneity with respect to fracture pattern, which makes their classification and preoperative planning particularly problematic. Articular depression, comminution, diaphyseal extension, and ligamentous and other soft tissue injuries are key elements that have to be considered. Thus, several classification systems have been proposed in order to facilitate the description of fractures, development of specific treatment algorithms, and comparison of prognosis [1-6]. Comprehensiveness, accuracy, and reliability are only some of the features that should characterize an ideal classification system [7]. The Schatzker classification has traditionally been the cornerstone and constitutes the guide of surgical planning [8]. More specifically, the fractures are divided according to the location (medial plateau, lateral plateau, and bicondylar), pattern (split and depressed), and their extension to the metadiaphyseal area. Similarly, the AO Foundation-Orthopaedic Trauma Association (AO-OTA) classification system relies on the same rationale and matches significantly the Schatzker classification, while encompassing some additional categories. However, both of them are based only on one plane and fail to identify coronal fracture lines [9,10]. Thus, the Luo “three-column classification” (medial, lateral, and posterior) was proposed, which takes into consideration only the CT axial view. The aim of this study was to assess the interobserver agreement of the aforementioned classification systems (Schatzker, AO-OTA, and Luo) and investigate the impact of multiplane CT scan on their reliability.

This article was previously presented as a meeting abstract at the EFORT Congress in June 2021.

## Materials and methods

Study design

Twenty-five cases of tibial plateau fractures were selected out of a large series of consecutive patients over a 10-year period. Computer-based random selection was applied to eliminate the likelihood of intentionally choosing those cases, which are particularly difficult to classify (selection bias). Twelve raters (seven consultants and five senior trainees) were invited to classify these cases according to Schatzker, Luo, and AO-OTA and select between different treatment options. Each rater was provided with a comprehensive schematic illustration of the three classifications to minimize bias because of the inadequate knowledge of the classification systems. The raters were unaware of the identity of the patients or the treatment, which they had finally undergone. Initially, a plain anteroposterior (AP) X-ray was demonstrated to the raters, and they were asked to classify the fracture according to Schatzker and AO-OTA. Then, an axial CT image was presented, and they had to classify the fracture according to Luo. This procedure was applied for 25 cases consecutively. Next, the raters were provided with additional information based on the sagittal and coronal CT views of the same cases. In this phase, they were requested to reevaluate their initial answers with respect to Schatzker and AO classification.

Data collection

At the end of the procedure, each rater had to disclose his/her level of training (consultant versus trainee) before delivering the completed form blindly. Our primary outcome was the interobserver reliability of the Schatzker, AO-OTA, and Luo classifications based on the initial AP X-ray and axial CT. Secondary outcomes included the improvement or not in our primary outcome after providing additional CT images.

Statistical analysis

Interobserver reliability was assessed using the Fleiss kappa coefficient, which is a widely used measure of interobserver agreement among multiple raters. The Fleiss kappa calculates the degree of agreement over that which will be expected by chance. The interpretation of kappa values is based on the guidelines of Landis and Koch [11]: values between 0 and 0.20 represent slight agreement, values between 0.20 and 0.40 represent fair agreement, values between 0.40 and 0.60 represent moderate agreement, values between 0.60 and 0.80 represent substantial agreement, and values between 0.80 and 1 represent almost perfect agreement. Negative values indicate an agreement, which is lower than it would occur by chance. Intraobserver reliability (before and after assessing the CT scans) was evaluated using Cohen’s kappa coefficient, which is also a chance-corrected measure of agreement between two ratings. The 95% confidence intervals (CIs) were calculated, and statistical comparisons were made upon them with a level of significance set at p<0.05. The analysis was performed on SPSS version 23 (IBM Corporation, Armonk, NY, USA).

## Results

An overall fair inter-rater agreement was observed for the Schatzker classification based on the plain AP X-ray (k=0.361) (Table 1). With respect to fracture pattern, the agreement was higher for type VI fractures and lower for type I fractures (type VI: k=0.624; type I: k=0.189) (Table 2). After evaluating the three-dimensional (3D) plane CT views, the raters reconsidered their initial answers in 27.8% of the cases, which corresponds to a mean intraobserver kappa value of 0.636. Thus, the inter-rater agreement improved slightly (k=0.364) (Table 1), although not statistically significant. Similarly, a kappa value of 0.204 was calculated for the AO-OTA classification based on the plain X-rays, which improved significantly to 0.231 after assessing the CT scans (Table 1). Finally, the Luo classification achieved the highest scores among the three classification systems (k=0.498), but its inter-rater agreement can still be characterized as moderate (Table 3). No statistically significant improvement in intra- or interobserver agreement was observed for any classification even if only the consultants’ subgroup was included in the data analysis.

**Table 1:** Interobserver agreement for tibial plateau classification systems: before and after three-dimensional CT views.

			Schatzker	AO-OTA	Luo
Interobserver agreement	Before 3D plane CT views	Kappa value* (95% CI)	0.361 (0.338–0.385)	0.204 (0.186–0.222)	0.498 (0.466–0.531)
		Interpretation	Fair	Fair	Moderate
	After 3D plane CT views	Kappa value* (95% CI)	0.364 (0.336–0.391)	0.231 (0.21–0.251)	N/A
		Interpretation	Fair	Fair	N/A

**Table 2:** Interobserver agreement with respect to Schatzker stages based on AP X-ray and reevaluation after the 3D plane CT views. *Statistically significant changes in interobserver agreement at a level of p≤0.05

Schatzker stage	Interobserver agreement (kappa value)	
	Based on AP X-ray	After 3D plane CT views
I	0.189*	0.241*
II	0.406	0.374
III	0.284*	0.115*
IV	0.393	0.400
V	0.237	0.272
VI	0.624*	0.470*
Overall	0.361	0.364

**Table 3:** Interpretation of kappa values for Luo classification system.

Luo classification	Kappa value (95% CI)	Interpretation
0	0.336 (0.257–0.354)	Fair
1	0.506 (0.457–0.554)	Moderate
2	0.462 (0.414–0.511)	Moderate
3	0.584 (0.535–0.632)	Moderate
Overall	0.498 (0.466–0.531)	Moderate

## Discussion

A classification system should meet the criteria proposed by Bland and Altman [12]: face and content validity, accuracy and reliability, and the prognostic value of fracture categories. The reliability of a system refers to its ability to produce consistent results either among different users or by a single user at different times. On the other hand, being accurate requires that the produced results are indeed correct. Thus, reliability alone does not ensure validity, if most of the raters agree on an incorrect answer. While reliability and accuracy can be evaluated by standard methodological tools, the third criterion is much more complex to assess. It represents the actual relationship between fracture categorization and expected outcomes in the context of a specific treatment algorithm [13].

Tibial plateau classification has been the focus of interest of many researchers with at least 38 classification systems being available in the literature [6]. However, the majority of them did not meet wide clinical acceptance. Fragment location, displacement, articular surface depression, and meta-diaphyseal extension are the basic factors that most of them incorporate. To our knowledge, ligamentous injuries are not addressed preoperatively in any of these classifications.

Among them, the Schatzker, AO-OTA, and Luo classification systems are probably the most widely used, both in clinical practice and for research purposes. We intended to investigate whether these systems actually provide a valid tool in terms of reliability. Our findings suggest that their reliability can be characterized as fair to moderate, which is more or less consistent with previous data. However, significant heterogeneity of data is documented in the literature, mainly due to different study designs and different raters’ expertise. Specifically, Zhu et al. calculated a kappa value of 0.567 (moderate) and 0.766 (substantial) for the Schatzker and Luo classifications, respectively [14]. Walton et al. showed only fair interobserver agreement for the Schatzker classification and moderate agreement for the AO-OTA classification [15]. Similarly, the findings of Mellema et al. suggest an overall fair interobserver reliability both for the Schatzker and Luo classification systems (Schatzker: k=0.32; Luo: k=0.28) [16].

Each classification system should be properly evaluated based on this particular imaging modality, which had been originally used to define this system. Nevertheless, we tried to investigate whether improved imaging could improve the reliability of the system. The introduction of three-dimensional CT has been reported to generally increase the interobserver reliability of each classification system [17-19]. The results of our study also show that the 3D plane CT slightly improves the interobserver agreement, but not to a remarkable degree. This finding suggests that the high variability among the raters actually reflects the weaknesses of the classification systems themselves, rather than disagreement on the fracture pattern due to unsatisfactory imaging.

The limitations of this study merit to be mentioned. First of all, only the most accepted classification systems were included in the study. Second, only one image of the 3D CT was chosen for each plane and presented to the raters. However, it was selected as being the most representative of the fracture pattern by an independent reviewer. The interpretation of the kappa coefficient should be made cautiously since it relies on relatively arbitrary guidelines. Furthermore, the kappa statistic is considered an overly conservative index of agreement. The relatively limited sample size is another weakness. Finally, our study evaluates only the reliability and not the accuracy or the prognostic value of the classification systems, which are equally important markers of validity.

## Conclusions

In conclusion, both the AO-OTA and the Schatzker classification systems failed to achieve a substantial agreement among the raters. The Luo classification performed better and seems to be more treatment-oriented. Future research should focus on classification systems that facilitate the establishment of an associated treatment algorithm, rather than being purely descriptive. Ligamentous and meniscal injuries, even if diagnosed during the operation, should also be taken into account, as they adversely affect the clinical outcomes.
